# Supplementary material for: Continuous treatment with abemaciclib leads to sustained and efficient inhibition of breast cancer cell proliferation
Source: Oncotarget. 2022 Jul 2;13:864–75. doi: 10.18632/oncotarget.28249 (PMC9255995; doi:10.18632/oncotarget.28249)
Supplement: Supplementary file 2 [file oncotarget-13-28249-s002.docx]

**Supplemental Table S3.** **Breast cancer cell lines.**

| **cell line** | **ER** | **HER2** | **TNBC** | ***BRAC1/2* ^(h)^** | ***RB1 mutations* ^(h)^** | **Rb expression ^(i)^** | **PIK3CA *mutations* ^(h)^** | **subtype** | **source** |
| --- | --- | --- | --- | --- | --- | --- | --- | --- | --- |
| BT-474 | ER+ | HER2+ |  | MUT BRCA2 (substitution-nonsense) | WT | + | MUT PIK3CA (substitution-nonsense) | luminal B | a,b, f, h, i |
| HCC1419 | ER+ | HER2+ |  | WT | WT | + | WT | luminal B | b, f, h, i, |
| MDA-MB-361 | ER+ | HER2+ |  | MUT BRCA2 (substitution-missense) | WT | + | MUT PIK3CA (substitution-nonsense) | luminal B | b, f, h, i |
| UACC-3133 | ER+ | HER2+ |  | NA | NA | + | NA | luminal B | h, i, |
| ZR-75-30 | ER+ | HER2+ |  | MUT BRCA2 (substitution-missense) | WT | + | WT | luminal B | b, f, h, i |
| CAMA-1 | ER+ | HER2- |  | WT | WT | + | WT | luminal B | b, f, h, i |
| EFM-19 | ER+ | HER2- |  | WT | WT | + | MUT PIK3CA (substitution-nonsense) | Luminal A | b,f, h, i |
| MCF-7 | ER+ | HER2- |  | NA | NA | + | MUT PIK3CA (substitution-nonsense) | Luminal A | a, f, h, i |
| MDA-MB-134-VI | ER+ | HER2- |  | NA | NA | + | WT | Luminal A | b,f, h, i |
| MDA-MB-175-VII | ER+ | HER2- |  | WT | WT | + | WT | Luminal A | b,f, h, i |
| MDA-MB-415 | ER+ | HER2- |  | WT | WT | + | WT | Luminal A | b, f, h, i |
| T-47D | ER+ | HER2- |  | NA | NA | + | MUT PIK3CA (substitution-nonsense) | Luminal A | b, f,h, i |
| ZR-75-1 | ER+ | HER2- |  | NA | NA | + | WT | Luminal A | b, f,h, i |
| UACC-812 | ER- | HER2+ |  | WT | WT | + | WT | HER2 | c, f, h i |
| AU565 | ER- | HER2+ |  | WT | WT | + | WT | HER2 | f, h, i, l |
| HCC1569 | ER- | HER2+ |  | MUT BRCA2 (substitution-missense) | WT | + | MUT PIK3CA (substitution-nonsense) | HER2 /post-EMT | b, f, h, i |
| HCC1954 | ER- | HER2+ |  | MUT BRCA1 (frameshift deletion) | WT | + | MUT PIK3CA (substitution-nonsense) | HER2/Basal | b, f, h, i |
| HCC202 | ER- | HER2+ |  | WT | WT | + | MUT PIK3CA (substitution-nonsense) | HER2 | b, f, h, i |
| HCC2218 | ER- | HER2+ |  | WT | WT | + | WT | HER2 | f, h, i, k |
| MDA-MB-453 | ER- | HER2+ |  | WT | WT | + | MUT PIK3CA (substitution-nonsense) | HER2 | a, e, f, h, i |
| SK-BR-3 | ER- | HER2+ |  | NA | NA | + | WT | HER2 | a, b, f, e, h, i |
| UACC-3199 | ER- | HER2+ |  | NA | NA | **-** | NA | HER2 | f, h |
| UACC-893 | ER- | HER2+ |  | WT | WT | + | MUT PIK3CA (substitution-nonsense) | HER2 | b, f, h, i |
| BT-20 | ER- | HER2- | TNBC | MUT BRCA2 (substitution-missense) | MUT (substitution missense) | + | MUT PIK3CA (substitution-nonsense) | basal | b, f, h, i |
| BT-549 | ER- | HER2- | TNBC | WT | WT | - | MUT PIK3CA (substitution-nonsense) | basal Claudin-Low | e, f, h, i |
| DU-4475 | ER- | HER2- | TNBC | WT | WT | - | WT | luminal | f, h, i |
| HCC1143 | ER- | HER2- | TNBC | WT | WT | + | WT | basal | b, f, h, i |
| HCC1187 | ER- | HER2- | TNBC | WT | WT | - | WT | basal | b, f, h, i |
| HCC1395 | ER- | HER2- | TNBC | MUT BRCA2 (substitution-nonsense) | WT | + | WT | basal | g, h, i |
| HCC1806 | ER- | HER2- | TNBC | WT | WT | + | WT | basal | b, f, h, i, |
| HCC1937 | ER- | HER2- | TNBC | WT | MUT | + | WT | basal | b, f, j, g, h |
| HCC38 | ER- | HER2- | TNBC | WT | WT | + | MUT PIK3CA (substitution-nonsense) | basal | b, f, h, i |
| HCC70 | ER- | HER2- | TNBC | WT | MUT (deletion in frame) | - | WT | basal | b, f, h, i |
| Hs-578-T | ER- | HER2- | TNBC | WT | WT | + | WT | basal Claudin-Low/ post-EMT | a,b, f,h, i |
| MDA-MB-157 | ER- | HER2- | TNBC | WT | WT | + | WT | post-EMT | b, f, h, i |
| MDA-MB-231 | ER- | HER2- | TNBC | WT | WT | + | WT | basal Claudin-Low/ post-EMT | a,b, f,h, i |
| UACC-2087 | ER- | HER2- | TNBC | NA | NA | + | NA |  | f, h, i |

^a^ Breast Cancer: Basic and Clinical Research 2010:4 35–41; ^b^ Breast Cancer Res Treat (2007) 105:319–326; ^c^ Breast Cancer Res. 2011; 13(6): R121.; ^d^ Mol Cancer Ther. 2018 May;17(5):897-907; ^e^ Holliday, D.L., Speirs, V. Choosing the right cell line for breast cancer research. Breast Cancer Res 13, 215 (2011); ^f^ J Cancer. 2017; 8 (16): 3131–3141; ^g^ Breast Dis. 2010; 32 (1-2): 35–48; ^h^ COSMIC.db (Nucleic Acids Research, Volume 47, Issue D1, 08 January 2019, Pages D941–D947); ^i^ internal data (WB); ^j^ Mol Cancer Ther; 16 (12) December 2017; ^k^ Breast Cancer Res 19, 65 (2017); ^l^ Am J Cancer Res. 2016; 6 (11): 2661–2678

ER: estrogen receptor; HER: human epithelial receptor; TNBC: triple-negative breast cancer

Signature for Rb expression in WB: (+) Visible band of Rb total and phosphoRb (Ser780); (-) Total Rb band is not visible or barely visible with no sign of Rb phosphorylation pRb (Ser780)
